# Cellular Conditions Responsible for Methylmercury-Mediated Neurotoxicity

**DOI:** 10.3390/ijms23137218

**Published:** 2022-06-29

**Authors:** Masatake Fujimura, Fusako Usuki

**Affiliations:** 1 Department of Basic Medical Sciences, National Institute for Minamata Disease, Kumamoto 867-0008, Japan; 2 Division of Neuroimmunology, Joint Research Center for Human Retrovirus Infection, Kagoshima University, Kagoshima 890-8544, Japan; fusuki@kufm.kagoshima-u.ac.jp

**Keywords:** methylmercury, site-specific neurotoxicity, redox ability, neural hyperactivation, mitogen-activated protein kinase cascade, Rho/ROCK signaling, ER stress, microglia

## Abstract

Methylmercury (MeHg) is a widely known environmental pollutant that causes severe neurotoxicity. MeHg-induced neurotoxicity depends on various cellular conditions, including differences in the characteristics of tissues and cells, exposure age (fetal, childhood, or adulthood), and exposure levels. Research has highlighted the importance of oxidative stress in the pathogenesis of MeHg-induced toxicity and the site- and cell-specific nature of MeHg-induced neurotoxicity. The cerebellar granule cells and deeper layer cerebrocortical neurons are vulnerable to MeHg. In contrast, the hippocampal neurons are resistant to MeHg, even at high mercury accumulation levels. This review summarizes the mechanisms underlying MeHg-mediated intracellular events that lead to site-specific neurotoxicity. Specifically, we discuss the mechanisms associated with the redox ability, neural outgrowth and synapse formation, cellular signaling pathways, epigenetics, and the inflammatory conditions of microglia.

## 1. Introduction

Methylmercury (MeHg) is a well-known neurotoxicant that causes serious and irreversible neurological dysfunction, depending on the cellular context and developmental phase. MeHg can pass through the blood–brain barrier, which blocks almost all toxic substances. MeHg causes lesions in both the central and peripheral nervous systems; these lesions are associated with the exposure concentration, the exposed age, and injured neuronal cell types, leading to characteristic neurological dysfunction. Minamata disease is MeHg-intoxicated neurological disorder with two characteristic clinical forms—fetal and adult. Fetal-type Minamata disease is caused by exposure to MeHg in utero and is characterized by extensive brain lesions leading to cerebral palsy-like clinical features with delayed psychomotor development [1]. Contrastingly, adult-type is caused by MeHg intoxication in adulthood and shows Hunter-Russell syndrome-like features, with the primary lesions involving the central nervous system (cerebellum, cerebrum, and dorsal root ganglia) and peripheral sensory nervous system [2,3]. Patients with this condition show neurological signs associated with site-specific pathological lesions.

To date, many studies on the pathogenic processes of MeHg exposure have been conducted using various cells and animal models. The above-mentioned pathological changes in MeHg toxicity do not directly correspond to the accumulation of mercury (Hg) in tissues. In a MeHg-exposed subacute rat model (fed 20 ppm MeHg in drinking water every day for 28 days), the most acute pathology was seen in the cerebellum with lower Hg concentrations than that in the liver and kidneys, which displayed fewer pathological changes despite the high Hg content [4]. Furthermore, it is known that the MeHg-induced pathological changes differ among animal species. In a MeHg-exposed mouse model, the mice showed pathological changes in the cerebra, but not in the cerebellums, which is different from the changes observed in rats. In animal MeHg exposure experiments, attention must be paid to the selection of animals for experimental purposes including their target organs. Site-and cell-specific pathological changes are characteristic of MeHg toxicity. It has been demonstrated that the deep layers of cerebrocortical neurons, cerebellar granule cells, dorsal root ganglia, and dorsal root nerves are all susceptible to MeHg, while the shallow layers of cerebrocortical neurons, cerebellar Purkinje cells, ventral root nerve, and the hippocampal neurons are resistant [5,6,7,8]. MeHg-mediated neurotoxicity depends on various cellular conditions and cellular responses, which play crucial roles in the development of MeHg-induced pathogenic changes. Many researchers have noted the importance of oxidative stress in the pathogenesis of MeHg-induced toxicity. Various MeHg intoxication studies have reported the early incidence of reactive oxygen species (ROS) induction and that the elimination of ROS can prevent MeHg cytotoxicity in vitro and in vivo. Recent studies have clarified that the molecular mechanisms underlying MeHg-mediated cellular damage involve MeHg-mediated oxidative stress [9].

Since different cellular redox abilities and cellular signaling systems play important roles in MeHg neurotoxicity, we focus on the cellular conditions responsible for MeHg-mediated site- and cell-specific pathological changes in this review.

## 2. MeHg-Mediated Neurotoxicity in Cerebellum

### 2.1. Involvement of Local Redox Ability in Site-Specific Cerebellar Neurotoxicity

The cerebellum is one of the main lesion sites of MeHg-mediated neurotoxicity, and these lesions are known to be site-specific. The cerebellar cortex consists of three geometrically ordered layers. The innermost layer is densely packed with numerous small neurons called granule cells. The middle layer contains Purkinje cells, and the outermost molecular layer contains stellate and basket cells. Among these cerebellar neurons, cerebellar granule cells, which are known to be excitatory cells [10], are the most vulnerable to MeHg in humans [11]. 

These site-specific cerebellar lesions can be produced in MeHg-intoxicated animal models [5,12]. Treatment with antioxidants can prevent MeHg-induced neurotoxicity in rats, which suggests that oxidative stress plays a role in MeHg-mediated neural toxicity [4,13]. Antioxidative enzymes, such as copper, zinc superoxide dismutase (Cu, Zn-SOD), manganese superoxide dismutase (Mn-SOD), glutathione peroxidase 1 (GPx1), thioredoxin reductase 1 (TRxR1), and catalase, are intrinsic antioxidants that prevent cellular oxidative stress. A previous study reported whether these intrinsic antioxidants play a role in causing these site-specific cerebellar lesions [5]. In this study, each cell type was isolated from the three cerebellar layers using a microdissection system and performed real-time PCR analysis of antioxidative enzyme-encoding genes. Results demonstrated that the basal abilities of antioxidative enzymes differed among these three cerebellar layers of neurons. Cerebellar granule cells exhibited much lower in situ expression of *Mn-SOD*, *GPx1*, and *TRxR1* mRNAs than cells in the cerebellar molecular layer or the Purkinje cells. Furthermore, immunohistochemistry showed stronger expression of Mn-SOD, GPx1, and TRxR1 in Purkinje cells and molecular layers of neurons than in granule cell layers, which was consistent with the mRNA expression pattern. Since Mn-SOD, GPx1, and TRxR1 are critical factors in protecting cells against MeHg-induced oxidative stress [9,14], these results suggest that low basal expression of these antioxidative enzymes contributes to MeHg-mediated site-specific neurotoxicity in the cerebellum.

### 2.2. Involvement of Disruption of Intracellular Ca^2+^ Regulation in Cerebellar Neurotoxicity

MeHg causes severe disruption of intracellular Ca^2+^ regulation, which contributes to cell death of primary cultures of rat cerebellar granule neurons [15]. Cerebellar granule neurons appear to be more susceptible to MeHg-induced dysregulation of divalent cation homeostasis and subsequent cell death when compared to purkinje neurons [16]. It has been demonstrated that MeHg-induced Ca^2+^ dysregulation and subsequent neurotoxicity of cerebellar granule cells are caused by the activation of M_3_ muscarinic receptor-linked pathway [15]. Cerebellar granule cells express the highest density of M_3_ muscarinic receptors [17], suggesting that the specific localization of M_3_ muscarinic receptors also contributes to MeHg-mediated site-specific neurotoxicity. The recent study added the findings that multiple sources of Ca^2+^ contribute to an increased frequency of MeHg-induced spontaneous inhibitory synaptic responses in rat cerebellar slices [18].

### 2.3. Impairment of Neurite Outgrowth in the Developing Cerebellum

The brains of human fetuses and neonates are extremely vulnerable to MeHg even at low exposure levels [1]. The previous report demonstrated that exposure to low levels of MeHg (5 ppm in drinking water) during the prenatal and lactation stages reduces the postnatal expression of pre- and post-synaptic proteins through reduced neurite outgrowth in the rat cerebellum; the newborn rats exhibited impaired motor coordination, while any neurotoxicity was not observed in maternal brain [19]. These results suggested that MeHg disturbs synaptic development and impairs motor coordination. Studies on the underlying mechanism demonstrated that 5 ppm MeHg exposure from gestation days 1 to 20 suppressed the tropomyosin receptor kinase (Trk), which disturbed the neuronal differentiation and synapse formation in fetal rats. In addition, downregulation of eukaryotic elongation factor 1A1 (eEF1A1) was observed on postnatal day (PND) 1 [20]. The role of eEF1A1 was confirmed via a knockdown study using differentiating PC12 cells. Knockdown of eEF1A1 in differentiating PC12 cells caused impaired neurite outgrowth and synaptic protein expression, similar to the results of prenatal exposure to MeHg in the developing cerebellum. Taken together, MeHg downregulates the TrkA pathway and eEF1A, thereby impairing synaptic formation in the developing cerebellum.

Recently, it has been suggested that the toxic effects of chemicals on fetal development may be related to epigenetic alterations, such as DNA methylation and histone modification. The epigenetic effects of low-level MeHg exposure on neuronal development were evaluated using in vitro and in vivo experiments [21]. The results showed reduced acetylated histone H3 (AcH3) levels and increased histone deacetylase (HDAC) 3 and HDAC6 levels, both in vitro and in vivo, with the MeHg-mediated suppression of neurite outgrowth. In addition, increased levels of DNA methylation and DNA methyltransferase 1 (DNMT1) were observed. These results suggest that MeHg-induced epigenetic changes may also contribute to the impairment of neurite outgrowth in the developing cerebellum.

### 2.4. Cerebellar Synaptic and Neuritic Remodeling in Pregnant Rats

Although it is well established that fetuses are extremely susceptible to MeHg toxicity [1], little is known about the effects of a very a low-dose of MeHg exposure on maternal neurons. The recent study demonstrated that pregnant rats exposed to very low levels of MeHg (1 ppm in drinking water) showed temporal cerebellar synaptic and neuritic remodeling during the perinatal period between gestational day 20 and PND 1, while vehicle-exposed pregnant rats exhibited no change [22]. These changes were transient and were reversed by PND 21. To elucidate the mechanisms underlying the perinatal changes observed in MeHg-exposed pregnant rats, the proteins related to synapse formation and neurite outgrowth were assessed. The result identified suppression of the TrkA pathway and reduced expression of the activity-regulated cytoskeleton-associated protein (Arc). Furthermore, an increase in plasma corticosterone levels and a decrease in estradiol levels were observed in MeHg-exposed pregnant rats compared with those in the vehicle-exposed pregnant control rats. The changes in TrkA pathway activity, Arc expression, and plasma hormone levels were normalized in parallel with restoration of synaptic and neuritic changes. These results suggest that very low levels of MeHg affect perinatal synaptic and neuritic remodeling in the maternal cerebellum through modulation of the TrkA pathway and Arc expression, which may be caused by MeHg-induced maternal hormonal changes. Although the importance of this phenomenon has not been so far clear enough, it may be involved in the biological defense response to MeHg toxicity in the mother’s brain [23].

## 3. MeHg-Mediated Neurotoxicity in the Cerebral Cortex

### 3.1. Involvement of Local Redox Ability in Site-Specific Neurodegeneration

The cerebral cortex consists of a six-layered neocortex, numbered I to VI, from the outermost layer I to the innermost layer VI. Autopsy studies of adult-type Minamata disease have shown that MeHg-induced cerebral lesions are localized to the deeper layers of the cerebral cortex, especially layer IV [2]. 

Such site-specific cerebral lesions can be reproduced in a MeHg-intoxicated mouse model [6,24]. The cause of this site-specific MeHg neurotoxicity in model mice was investigated with a focus on in situ antioxidative systems because of their critical role in MeHg intoxication [6,25,26]. In situ analyses of antioxidative enzyme expression were performed in laser-microdissected cerebrocortical neurons of shallower layers (sl-CCNs) and deeper layers (dl-CCNs). RT-qPCR analyses showed lower basal mRNA expression levels of *Mn-SOD* and *GPx1* in the dl-CCNs than in the sl-CNNs. Subsequently, antioxidative enzymes activity analyses for the separated cerebral cortex showed that Mn-SOD and GPx1 activities were lower in dl-CCNs than in sl-CCNs. 

These results suggest that low basal expression of these antioxidative enzymes may contribute to MeHg-mediated site-specific neurotoxicity in the cerebral cortex, which is similar to the layer-specific sensitivity seen in the cerebellum as already mentioned in Section 2.1 in this review.

### 3.2. MeHg-Mediated Neural Hyperactivation in the Deep Layers of Cerebrocortical Neurons

Cerebrocortical neurons of layer IV are mainly composed of excitatory cells, similar to the cerebellar granule cells. This suggests that excitatory cells respond readily to MeHg-induced neural hyperactivation, leading to neuronal damage. Genes that are activated transiently and rapidly in response to various cellular stimuli are referred to as immediate early genes (IEGs). Among IEGs, c-fos and brain-derived neurotrophic factor (BDNF) are potent markers of neural activity. In particular, c-fos has been extensively used to identify site-specific neural activity in the brain [27,28]. 

The relation between the MeHg-induced changes in site-specific expression of IEGs and neural activity has been investigated in a MeHg-intoxicated mouse model that showed neurodegeneration in dl-CCNs, especially in layer IV. Immunohistochemical and Western blot analyses demonstrated that site-specific upregulation of c-fos and BDNF in dl-CCNs preceded neuropathological changes in the cerebral somatosensory cortex [24]. Western blot results showed that c-fos and BDNF expressed in parallel with the induction of cAMP response element-binding protein (CREB) via phosphorylation, a regulator of c-fos and BDNF. These changes were triggered by activation of the p44/42 MAPK, p38 MAPK, and cAMP-dependent protein kinase (PKA) pathways. This expression of c-fos and BDNF through activation of the MAPK and PKA/CREB pathways was not observed in the hippocampus or cerebellum, where neuronal degeneration was never observed in mice despite MeHg accumulation. MeHg-induced oxidative stress activates the MAPK pathway, leading to various cellular stress responses [24]. Taken together, these findings suggest that the MeHg-induced cerebrocortical neuronal degeneration was caused by site-specific neural hyperactivity, which was triggered by activation of the MAPK and PKA/CREB pathways, followed by c-fos and BDNF upregulation. 

Further investigation of MeHg-induced oxidative stress and the subsequent signaling pathways causing cerebrocortical neural hyperactivity and cell death was performed using *all*-*trans* retinoic acid-differentiated SH-SY5Y cells. These cells showed neuron-like morphological changes and the expression of neurons/synapse markers for cerebrocortical neurons [29]. Time-course studies clarified that MeHg-induced upregulation of c-fos preceded neuronal cell death, which was similar to the result observed in the cerebra of MeHg-intoxicated mice. The SH-SY5Y model showed that early induction of oxidative stress was followed by the activation of p44/42 MAPK and p38 MAPK and an increase in CREB expression. The antioxidative drugs Trolox and Edarabone significantly suppressed MeHg-induced oxidative stress, the p38 MAPK-CREB pathway, and neuronal cell death. Furthermore, treatment with SB203580, a specific inhibitor of p38 MAPK, significantly blocked the regulation of c-fos and neuronal cell death, whereas treatment with the p44/p42 MAPK inhibitors PD98059 and U0126 did not [29]. These results suggest that the MeHg-induced oxidative stress and subsequent activation of the p38 MAPK-CREB pathway contributes to neuronal hyperactivity and subsequent neuronal cell death. 

### 3.3. Role of the Rho-ROCK Signaling Pathway in MeHg-Mediated Axonal Degeneration

It has been reported that the ROCK signaling pathway is involved in various biological reactions such as neural axonal degeneration, microglial activation, and apoptotic cell death [30]. ROCK is expressed as two homologs, ROCK1 and ROCK2, which share similar structures and functions. In the brain, it has been reported that ROCK1 is distributed in glia, whereas ROCK2 in neurons [31]. 

**Figure 1:**
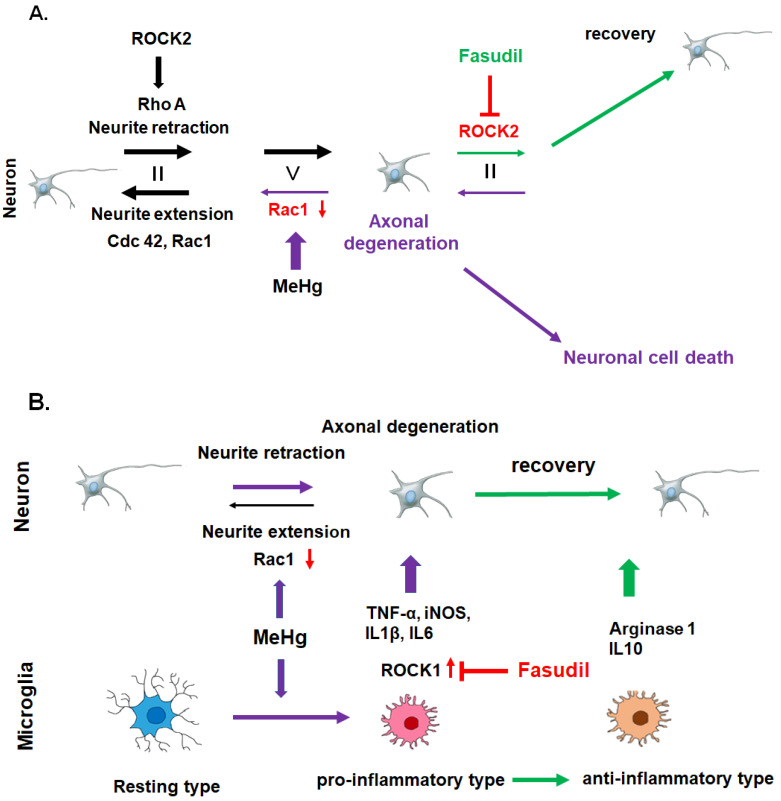
MeHg-mediated axonal degeneration is caused by a neurite outgrowth/contraction imbalance, and the effects are reversed by fasudil treatment. There are two mechanisms behind the therapeutic effect of fasudil on MeHg-induced neurodegeneration in the subacute phase (**A**) and in the chronic phase (**B**) in a MeHg-intoxicated rat model. (**A**) MeHg-induced downregulation of Rac1 causes a neurite outgrowth/contraction imbalance and axonal degeneration. ROCK2 inhibition by fasudil modifies the neurite outgrowth/contraction imbalance and protects cortical neurons from axonal degeneration and apoptotic neuronal cell death. (**B**) Fasudil suppresses the ROCK1 activity of invaded microglia, followed by downregulation of pro-inflammatory factors, including TNFα, iNOS, IL-1β, and IL-6, and upregulation of the anti-inflammatory factors arginase1 and IL-10. The Rho/ROCK pathway of microglia is essential for the chronic phase of MeHg-induced cerebrocortical neurodegeneration.

Neurite degeneration prior to neuronal cell death from MeHg exposure has been demonstrated in studies with cultured cerebral cortical neurons [20,32,33]. Previous studies demonstrated that the Rho-ROCK signaling pathway plays a role in this phenomenon both in vitro and in vivo [32,33,34,35]. Rho family proteins, including Rho, Ras-related C3 botulinum toxin substrate (Rac), and cell division control protein 42 homolog (Cdc42), are associated with neurite regulation and apoptotic cell death in neurons. Among the Rho family of proteins, Rac1 and Cdc42 are associated with neurite extension. In contrast, neurite retraction is promoted by RhoA as well as ROCK1 and 2, which are key effectors of RhoA [36]. Using cultured cerebrocortical neurons, we showed that MeHg-induced downregulation of Rac1, a protein related to neurite outgrowth, is the first event that triggers axonal degeneration and subsequent apoptotic neuronal cell death [32]. In contrast, the expression of RhoA, which is associated with neurite contraction, is unaffected by MeHg exposure. These results suggested that disturbances in the Rho/ROCK pathway result in a neurite outgrowth/contraction imbalance, which leads to MeHg-induced axonal degeneration. Several endogenous inhibitors, including myelin-related glycoproteins, NOGO, and oligodendrocyte-myelin glycoproteins, have been reported to interfere with axonal regeneration in the central nervous system (CNS) via the Rho/ROCK pathway [37]. It has been reported that modifiers of the Rho/ROCK pathway, including the Rho inhibitor C3 toxin, and the two ROCK inhibitors, fasudil and Y-27632 (selective ROCK1, 2 inhibitors), significantly protect cultured cortical neurons from MeHg-induced axonal degeneration and apoptotic neuronal cell death [32]. Furthermore, in vivo treatment with fasudil partially prevented pathological changes in the dorsal root ganglia, dorsal root nerve, and posterior column of the spinal cord by inhibiting ROCK2 in the subacute phase of a MeHg-intoxicated rat model [34]. Figure 1A summarizes the MeHg-mediated axonal degeneration caused by a neurite outgrowth/contraction imbalance and its recovery via Rho/ROCK pathway mediators.

Recently, much attention has been paid to blocking the effects of the Rho/ROCK signaling pathway on neurite outgrowth and apoptosis after CNS injury [38]. Regulation of the Rho/ROCK pathway may be a potential target to prevent MeHg-mediated axonal degeneration and apoptotic neuronal cell death. The recent study also demonstrated the therapeutic effect of fasudil in a chronic MeHg-intoxicated rat model, even after the onset of neuropathological changes and clinical signs [35]. Results demonstrated the recovery effect of fasudil on MeHg-induced axonal degeneration and clinical signs during the chronic neurotoxicity stage. The ROCK analysis study demonstrated that the therapeutic effect of fasudil was due to the transition of invaded microglia from a pro-inflammatory to an anti-inflammatory phenotype. Under MeHg exposure, the invaded microglia were pro-inflammatory and showed an increase in ROCK1 expression. Fasudil suppressed the ROCK activity of invaded microglia, which downregulated pro-inflammatory factors, including tumor necrosis factor α (TNFα), inducible nitric oxide synthetase (iNOS), IL-1β, and IL-6, and upregulated the anti-inflammatory factors arginase-1 and IL-10 [35]. These results suggest that changes in the levels of microglia-secreted cytokines have a recovery effect on MeHg-induced axonal degeneration and clinical signs. Figure 1B summarizes the mechanism of the in vivo therapeutic effect of fasudil in MeHg-induced neurodegeneration during the chronic phase of the MeHg-intoxicated rat model. Another study has corroborated the role of microglial ROCK1 in a chronic MeHg-induced mouse cerebrocortical neurodegeneration model [39].

### 3.4. MeHg-Mediated Activation of the c-Jun N-Terminal Kinase (JNK) Pathway Causes Neurodegeneration in Cerebrocortical Neurons

The microtubule-associated protein tau plays important roles in stabilizing microtubules and axonal transport in neurons. Although tau phosphorylation regulates axonal transport through its conformation under physiological conditions, under pathological conditions, hyperphosphorylation reduces the affinity of tau for microtubules and impairs axonal transport. Furthermore, hyperphosphorylated tau easily aggregates into paired helical filaments and neurofibrillary tangles, subsequently leading to neuronal death [40].

Tau hyperphosphorylation can be observed in the brains of mice exposed to MeHg (30 ppm MeHg in drinking water for eight weeks) [41]. Neuropathological studies have shown reduction in the number of neurons, neuronal necrosis, and apoptosis and increase in the number of astrocytes and microglia/macrophages in the cerebral cortex. Western blot analyses have demonstrated that MeHg exposure increases tau phosphorylation at Thr-205, Ser-396, and Ser-422 in the cerebral cortex, consistent with the phosphorylation patterns noted in Alzheimer’s disease and frontotemporal dementia. Furthermore, immunohistochemical analyses have shown that the distribution of tau-phosphorylated (Thr-205) neurons corresponds with areas showing considerable neuropathological changes. The activation of mitogen-activated protein kinase kinase 4 (MKK4) and JNK is particularly considered to be responsible for tau phosphorylation. Additionally, neuropathological changes and tau hyperphosphorylation are not detected in the hippocampus even if Hg concentration is twice that in the cerebral cortex. These results suggest that MeHg-induced tau phosphorylation is mainly triggered by the activation of JNK pathways and contributes to neuropathological changes in the cerebral cortex [41]. 

Meanwhile, JNK pathway has been reported to be related to autophagy induction and play an important role in neurotoxicity [42]. Beclin1 and vacuolar sorting protein 34 (Vps34), a class III phosphatidylinositol-3 kinase are known to play an essential role in the cellular process of autophagy [43]. A recent study demonstrated that MeHg-induced JNK activation promotes the interaction between Beclin1 and Vsp34 and autophagy induction in cultured rat cerebrocortical neurons [44]. Although it has been reported that autophagy plays a protective role in cadmium- or manganese-induced neurotoxicity [45,46], the impairment of lysosomal function by MeHg promoted autophagosome accumulation and neuronal cell death [44]. MeHg-induced autophagy via JNK/Vps34 complex pathway also plays a role in neurotoxicity in the cerebral cortex.

### 3.5. Role of Endoplasmic Reticulum (ER) Stress in MeHg-Mediated Cerebrocortical Neurotoxicity

The ER is a membranous organelle that specializes in the folding and post-translational maturation of proteins. The ER redox stage is linked to ER protein folding homeostasis. Disulfide bond formation in the ER lumen is highly sensitive to an altered redox balance, and the disruption of protein folding causes ER stress.

A previous study showed that the failure to protect cells from MeHg-mediated early oxidative stress triggers ER stress and apoptosis [47]. A subsequent study showed that pretreatment with the antioxidant Trolox significantly blocked the MeHg-induced ER stress, thereby preventing unfolded protein response (UPR) activation, mitochondrial dysfunction, and apoptosis in primary cultured cortical neurons [48]. These results suggest that MeHg induces neuronal apoptosis through ROS-mediated ER stress and mitochondrial apoptosis pathways. Furthermore, a recent study showed site- and cell type-specific ER stress in the MeHg-exposed brains of ER stress-activated indicator (ERAI) transgenic mice [49]. The transgenic ERAI was constructed by fusing the gene encoding a variant of the green fluorescent protein Venus as a reporter downstream of a partial sequence of the human X-box binding protein 1 (XBP1), including an ER stress-specific intron [50]. XBP1 is a transcription factor spliced by inositol-requiring protein 1 (IRE1), a stress sensor of the UPR. UPR is a cytoprotective pathway against ER stress. The transgenic mice with this pathway proteins present with an ERAI signal when the UPR is activated by ER stress. Spatiotemporal time-course analyses of ER stress using these ERAI-Venus mice demonstrated that MeHg exposure (30 ppm MeHg in drinking water) induced the largest ERAI fluorescence signal in the somatosensory cortex three weeks after the start of exposure. Dual immunohistochemical analyses showed that almost all ERAI signals were predominantly localized in the neurons exposed to MeHg for three weeks. Time-course analysis revealed a stronger ERAI signal in the early phase, suggesting this was the period when the UPR pathway was activated. Downstream signaling of the IRE1-XBP1 axis was also observed in the early phase. These results indicate that MeHg induces ER stress that activates the UPR pathway in cerebrocortical neuronal cells in the early phase after exposure.

## 4. MeHg-Mediated Neurotoxicity in the Primary Afferent Nervous System

Pain can be divided into nociceptive and neuropathic types. While nociceptive pain is caused by inflamed or damaged tissues activating specialized pain sensors called nociceptors, neuropathic pain is caused by damage to or malfunction of the nervous system [51]. Neuropathological changes in the peripheral nerves, spinal cord dorsal roots, spinal cord dorsal ganglia, and cerebral cortex areas, corresponding to damaged sensory systems, have been reported in human and animal MeHg toxicity [3,52,53]. Mercuric compounds have been reported to cause neuropathic pain in humans [54,55], which suggests that the MeHg-induced neuropathological changes in primary afferent nerves may also trigger neuropathic pain. 

The recent study demonstrated the pathophysiological mechanism of MeHg-mediated neuropathic pain (hyperalgesia/allodynia) using a rat model of MeHg exposure (20 ppm in drinking water for three weeks) [56]. Histopathological studies using these rats demonstrated axonal degeneration in the primary afferent nervous system (peripheral sural nerve, dorsal root ganglion, dorsal root nerve, and dorsal column of the spinal cord), while no neuropathological changes were observed in the somatosensory cortex and thalamus [53,56]. The rats showed hyperalgesia/allodynia, evidenced by a significant decrease in the threshold of mechanical pain evaluated using an algometer with calibrated forceps [56]. Immunohistochemistry also revealed the accumulation of activated microglia in the dorsal root nerve, dorsal column, and dorsal horn of the spinal cord. An increase in the levels of inflammotoxic and inflammatory cytokines (TNFα, Il-1β, and IL-6), which are related to Iba1-positive inflammatory microglia, and phospho-CRE binding protein (CREB), which is related to neuronal activation, was revealed by Western blot analysis. These results suggest that dorsal horn neuronal activation is mediated by microglia-excreted inflammatory factors and that these changes alter nociceptive transmission within the spinal cord. Furthermore, analyses of the cerebral cortex demonstrated increased expression of phospho-CREB and astrocyte-released thrombospondin-1, which are important factors for excitatory synapse formation, specifically in the somatosensory cortical area. The expression of pre- and post-synaptic markers also increased in this cortical area. These observations suggest that this new cortical circuit is specifically wired in the somatosensory cortex. MeHg-mediated neuropathic pain may be caused by neuronal activation with inflammatory microglia in the dorsal horn and new cortical circuit formation in the somatosensory cortex [56]. 

## 5. Conclusions

**Figure 2:**
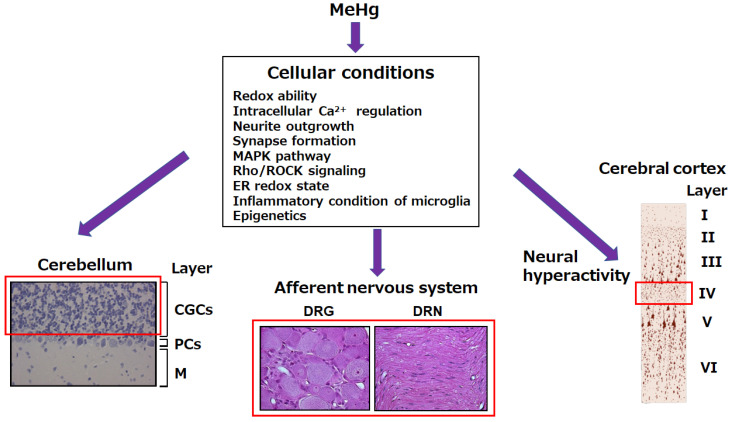
Cellular conditions responsible for MeHg-mediated site-specific neurotoxicity. MeHg-mediated site-specific neurotoxicity involves cerebellar granule cells, the primary afferent nervous system, and deep layers of cerebrocortical neurons (especially layer IV), which are surrounded by a red frame. The cellular conditions indicated here are responsible for the occurrence of MeHg-mediated site-specific neurotoxicity. CGCs, cerebellar granule cells; PCs, Purkinje cells; M, molecular; DRG, dorsal root ganglia; DRN, dorsal root nerve.

The pathological changes in MeHg toxicity do not directly correspond to the accumulation of Hg in the tissues, and various cellular conditions modify the MeHg-mediated toxicological processes. Oxidative stress in the early phase after exposure to MeHg plays a role in the pathogenesis of MeHg neurotoxicity. MeHg-mediated oxidative stress arises from the disruption of individual cellular redox systems and affects various cellular processes. The MeHg neurotoxicity involves neurite outgrowth and synapse formation, various cellular signaling pathways, the ER redox state, epigenetics, and the inflammation of microglia. An imbalance in cellular protective systems against MeHg toxicity leads to characteristic site-specific MeHg toxicity. To protect cells against MeHg-mediated cellular damage, an understanding of the mechanisms underlying MeHg toxicity is essential. A schematic overview of the cellular conditions that lead to MeHg-mediated site-specific neurotoxicity is presented in Figure 2.

## Data Availability

Not applicable.

## Abbreviations

MeHg	Methylmercury
Hg	Mercury
ROS	Reactive oxygen species
MAPK	Mitogen-activated protein kinase
Rho	Ras homolog gene
ROCK	Rho-associated coiled coil-forming protein kinase
Cu: Zn-SOD	Copper, zinc-superoxide dismutase
Mn-SOD	Manganese-superoxide dismutase
GPx1	Glutathione peroxidase 1
TRxR1	Thioredoxin reductase 1
PCR	Polymerase chain reaction
Trk	Tropomyosin receptor kinase
eEF1A1	Eukaryotic elongation factor 1A1
PND	Postnatal day
Arc	Activity-regulated cytoskeleton-associated protein
Sl-CCNs	shallower layers of cerebrocortical neurons
dl-CCNs	deep layers of cerebrocortical neurons
IEGs	Immediate early genes
BDNF	Brain-derived neurotrophic factor
CREB	cAMP response element binding protein
PKA	cAMP-dependent protein kinase
Rac	Ras-related C3 botulinum toxin substrate
Cdc42	Cell division control protein 42 homolog
CNS	Central nervous system
JNK	c-Jun N-terminal kinase
MKK4	Activated protein kinase kinase 4
Vsp34	Vacuolar sorting protein 34
ER	Endoplasmic reticulum
UPR	Unfolded protein response
ERAI	ER stress-activated indicator
XBP1	X-box binding protein 1
IRE1	Inositol-requiring protein 1
